# Supplementary material for: Relevance of Abnormal KCNN1 Expression and Osmotic Hypersensitivity in Ewing Sarcoma
Source: Cancers (Basel). 2022 Oct 1;14(19):4819. doi: 10.3390/cancers14194819 (PMC9564116; doi:10.3390/cancers14194819)
Supplement: Supplementary file 1 [file cancers-14-04819-s001.zip › cancers-1923824-supplementary.pdf]

Supplementary Materials

Relevance of abnormal KCNN1 expression and osmotic hypersensitivity in Ewing sarcoma

Sebastian Fuest[1‡], Christoph Post[1‡], Sebastian T. Balbach[2], Susanne Jabar[3], Ilka Neumann[1], Sandra Schimmelpfennig[1], Sarah Sargin[1], Elke Nass[4], Thomas Budde[4], Sareetha Kailayangiri[2], Bianca Altvater[2], Andreas Ranft[3], Wolfgang Hartmann[5], Uta Dirksen[3], Claudia Rössig[2], Albrecht Schwab[1], Zoltán Pethő[1*]

[1] Institute of Physiology II, University Münster, 48149 Münster, Germany
[2] University Children´s Hospital Münster, Department of Pediatric Hematology and Oncology, 48149, Germany
[3] Pediatrics III, University Hospital Essen, 45147 Essen, Germany
[4] Institute of Physiology I, University Münster, 48149 Münster, Germany
[5] Division of Translational Pathology, Gerhard-Domagk-Institute of Pathology, University Münster, 48149 Münster, Germany

* Correspondence: pethoe@uni-muenster.de

Supplementary materials:

1. **Supplementary Tables S1-S2**
2. **Supplementary Figures S1-S6**
3. **Supplementary Data**
4. **Supplementary Method**
5. **References**
6. **Uncropped Western Blots and PCR gels**

1. **Supplementary Tables:**

Table S1. Primers used in the experiments.

Gene (RefSeq ID)	Forward-Primer	Reverse-Primer	Source
GAPDH (NM_002046)	ACGACCCCTTCATTGAC CTCA	TTTGGCTCCACCCTTCAAGT G	[59]
YWHAZ (NM_003406)	CAACACATCCTATCAGA CTGGG	AATGTATCAAGTTCAGCAA TGGC	[60]
KCNMA1 (NM_002247)	ACGCTCAAGTACCTGTG GAC	AAGACAACCAGGACTCTGC C	De novo
KCNN1 (Exon 6-8) (NM_002248)	GCGCGTCTGCGAGAGGT A	GTACAGCCAGCTCCCATGAT	De novo
KCNN2 (NM_021614)	TCGTGGTCATGGTCATCG AG	CTCCAGTCATCTGCTCCATT GT	De novo
KCNN4 (NM_002250)	GGCCAAGCTTTACATGA ACACGCA	AAAGGTGCCCAGTGGCATTA ACAG	De novo
TRPC1 (NM_003304)	ACTGTGTAGGCATCTTCT GTGAACA	GGAGAAAATATACCAGAAC AAAGCAAA	[61]
TRPV4 (NM_021625.4)	TCCCATTCTTGCTGACCC AC	AGGGCTGTCTGACCTCGATA	De novo
TRPV6 (NM_018646)	GCCCTGAACAAGTTGCT CAAG	TGTCATAGAGGGCTGCTATG TGTAG	[61]
TRPC6 (NM_004621)	CCGCTGCCTTGCTACGG CTACT	TCTTCCGCACCACTGGGATG TT	[62]
TRMP2 (NM_003307)	ACGTGCTCATGGTGGAC TTC	AGGGTCATAGAAGAGCTGC C	[63]
TRPM7 (NM_017672)	CAGAAACCAAGCGCTTT CCT	AATTCAACGGCCAACTGACC	[64]
PIEZO1 (NM_001142864)	CAGGCCTATGAGGAGCT GTC	TTGTAGAGCTCCCGCTTCAT	[65]
PIEZO2 (NM_022068)	GCCCAACAAAGCCAGTT GAA	GGGCTGATGGTCCACAAAG A	[65]

CACNA1A (Cav2.1) (NM_001174080)	GTCTGGGGAAGAAGTGTCCG	GCTCCTCCCTTGGCAATCTT	De novo
CACNA1C (Cav1.2) (NM_199460)	TCCTCATCGTAATTGGCAGCA	TTCAGCTGGGTTTACCTCGG	De novo
CACNA1G (Cav3.1) (NM_018896)	ACACTTGGAACCGGCTTGAC	AGCACACGGACTGTCCTGA	[66]
CACNA1H (Cav3.1) (NM_021098)	CTTCTTCTGCCTCGGTCAGA	TGATTACCAGCATGCTCACG	[67]
CACNA1I (Cav3.1) (NM_021096)	TATGTCTGCCACATCCTGCG	ACCCTTAGTCTTCCCATGGTAGT	De novo
STIM1 (NM_003156)	GCTCCTCTGGGGACTCCT	CAATTCGGCAAAACTCTGCT	[68]
STIM2 (NM_020860)	GACGTCAGTATGCAGAACAGGA	TCAAATTCTTTTTCGGCCTTT	[68]
ORAI1 (NM_032790)	ACCTCGGCTCTGCTCTCC	GATCATGAGCGCAAACAGG	[68]
ORAI2 (NM_032831)	TACCTGAGCAGGGCCAAG	TGGCCACCATGGCAAAG	[68]
ORAI3 (NM_152288)	ACGTCTGCCTTGCTCTCG	GAGTGCAAAGAGGTGCACAG	[68]
KCNN1_Exon1	TCTGAGCTGGAGCCACG	CAGCTCTCCGAGTCCGTAGG	De novo
KCNN1_Exon1-4	TCTGAGCTGGAGCCACG	GCTGGGCTCCTGCACTGAC	De novo
KCNN1_Exon3-4	GAGCCCCGCAGGTCAGT	TTGGCCACTACCACCTGGAG	De novo
KCNN1_Exon5-6	ATGCCTCATCAGCCTCTCCA	CCACCATGAACAGCTGGATCT	De novo
KCNN1_Exon9-11	GCCATCCATCAGGCTCAGAA	AGGTCGGTAAGCGTGTTAGC	De novo
KCNN1_Exon12 (3'UTR)	CGCCACCAGACCCCTAAAT	ACTGTACAAGGCTTCCCGGA	De novo
EWSR1-FLI1	CAGCCTCCCACTAGTTACCC	GGTGAGGCCAGAATTCATG	[69]

Table S2. *KCNN1* expression in EwS does not correlate with clinical parameters of disease. *Contingency coefficient

KCNN1^{low} vs *KCNN1*^{high}		
correlation	r*	*P*
Primary tumor volume <200ml / >=200ml	0.02	0.89
Primary metastasis (no / yes)	0.02	.77
Event of relapse /secondary malignancy (no / yes)	0.03	.78
Time until relapse /secondary malignancy	0.22	.04
Histological response (good / poor)	0.08	.41
Overall survival (dead/alive)	0.00	.99
Gender (male / female)	0.03	.68
Age at diagnosis (< 15 years / >= 15 years)	0.00	1.0
Tumor location (axial vs non-axial)	0.02	.78

2. Supplementary Figures:

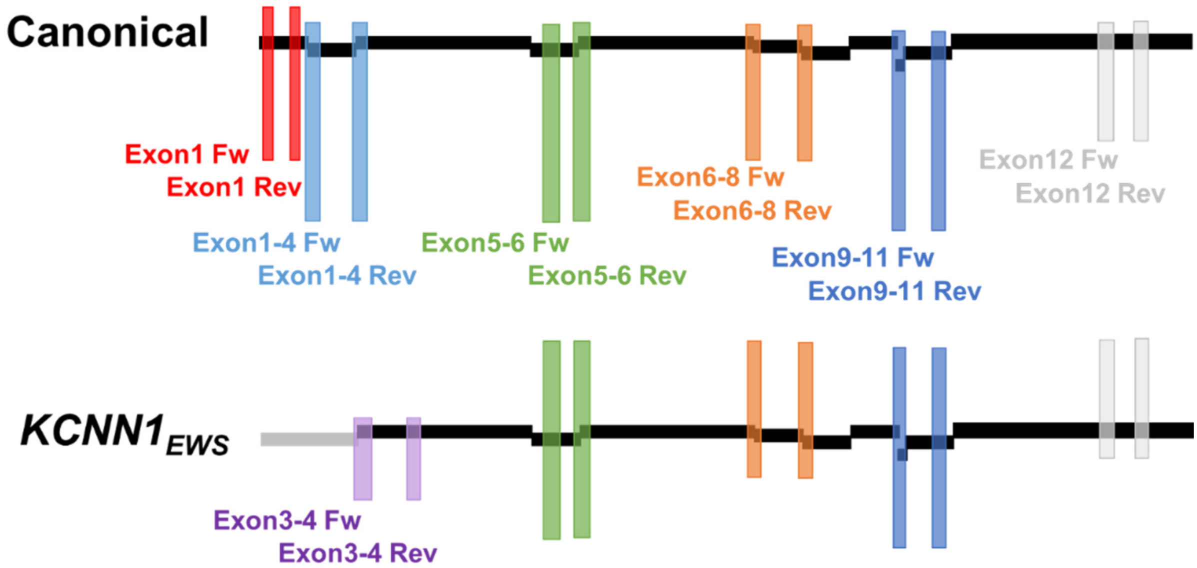

Figure S1. Primer binding sites for exon-detecting *KCNN1* primers.

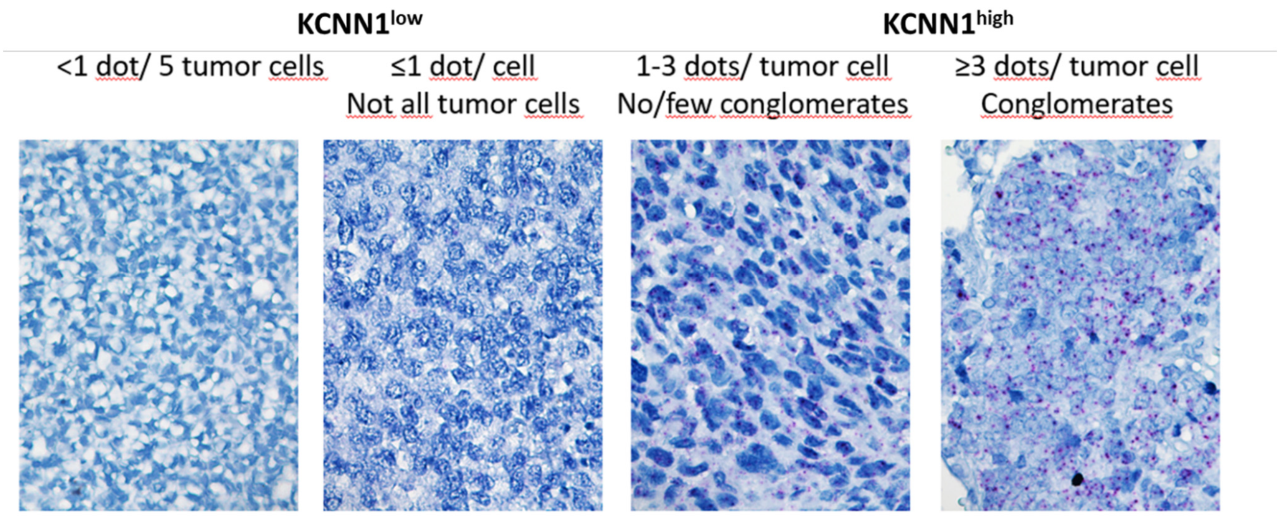

Figure S2. Scoring algorithm of RNAish. Representative images show criteria for classification into KCNN1low and KCNN1high groups, respectively, based on the number of RNAish signal (purple dots).

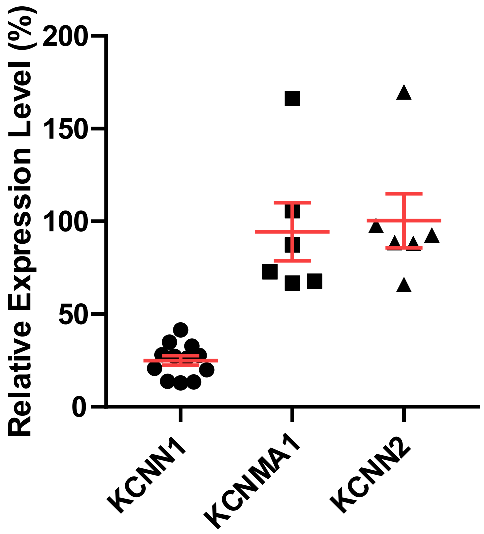

Figure S3. si*KCNN1* transfection interferes selectively with the expression of *KCNN1*. Scatter plot depicts relative gene expression of target genes compared to siScrambled transfection 24 following transfection (N=3, n_{KCNN1}=12, n_{KCNMA1}=6, n_{KCNN2}=6).

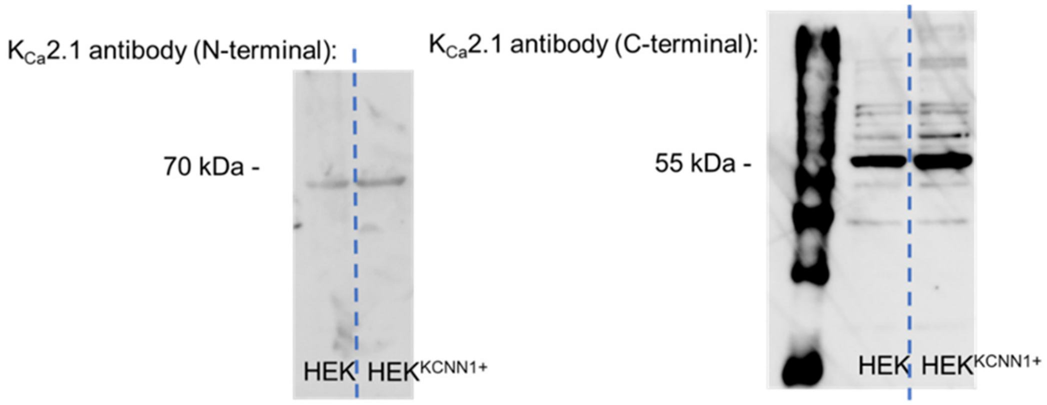

Figure S4. anti-*K_{Ca}2.1* antibodies do not specifically recognize the *K_{Ca}2.1 channel.*

Representative Western Blot chemiluminescent images from N=3 blots depict anti-*K_{Ca}2.1* signals against wild-type HEK293 cells (HEK) or HEK293 cells transfected with KCNN1 (HEK $^{KCNN1+}$), divided by blue dashed lines. The two types of available antibodies target peptide sequences from either the N- or the C-terminus of the protein.

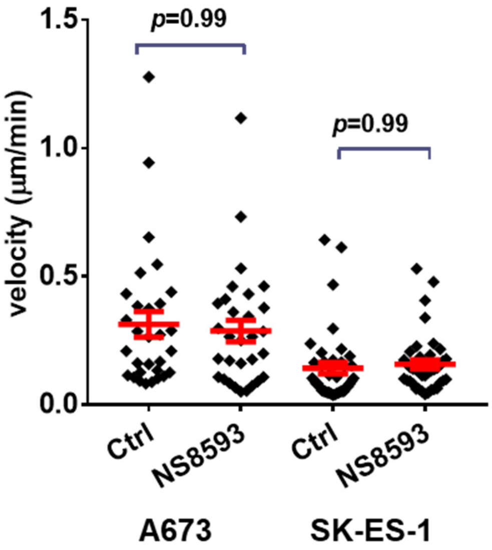

Figure S5. Membrane-permeable *K_{Ca}2.1* inhibitor NS8593 does not affect EwS cell migration.

Mean cell velocities from individual cells in the presence of 1 µM NS8593 or control (0.1% DMSO). A673: N=3, n=30, SK-ES-1: N=4, n=40

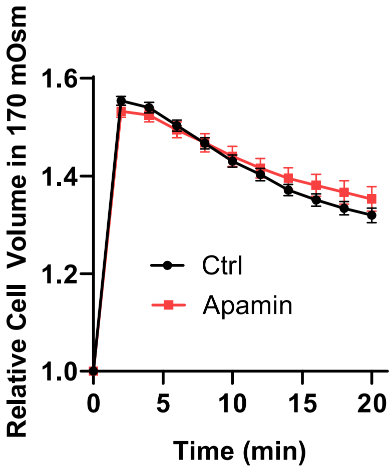

Figure S6. Apamin does not affect TC-71 cell regulatory volume decrease.

RVD performance of TC-71 cells treated with *siKCNN1* (red, N=4, n=32) or with scrambled siRNA (black, N=4, n=38), indicated by changes in their relative cell volume in a hypoos-motic solution (170 mOsm). The volume is normalized to their initial volume in an isosmotic solution

3. **Supplementary Data:**

KCNN1 transcripts derived from EWS cell lines using capillary sequencing. Forward and reverse sequencing primers are highlighted in yellow.

I. **NM_002248.5** (Homo sapiens potassium calcium-activated channel subfamily N member 1 (KCNN1), transcript variant 1, mRNA) **– 1809 bp**

CGAGGAGACCCCTGCACCCTAGGGTCAGTGCAGGAGCCCAGCCGCTGAGCCATGCCGGGCCCCG
GGCGGCCTGCAGCGAGCCCAACCCCTGCACCCAGGTAGTCATGAACAGCCACAGCTACAATGGC
AGCGTGGGGCGGCCGCTGGGCAGCGGGCCGGGCGCCCTGGGACGAGACCCTCCGGACCCTGAG
GCCGGCCACCCCCCACAACCCCCGCACAGCCCGGGCCTCCAGGTGGTAGTGGCCAAGAGTGAGC
CAGCCCGGCCCTCACCCGGCAGCCCCCGGGGGCAGCCCCAGGACCAGGACGATGACGAGGATG
ATGAGGAAGATGAGGCCGGCAGGCAGAGAGCCTCGGGGAAACCCTCAAATGTGGGCCACCGCC
TGGGCCACCGGCGGGCGCTCTTCGAGAAGCGGAAGCGCCTCAGCGACTATGCCCTCATTTTCGGC
ATGTTTGGCATCGTCGTCATGGTGACGGAGACCGAGCTGTCCTGGGGGGTGTACACCAAGGAGTC
TCTGTACTCATTCGCACTCAAATGCCTCATCAGCCTCTCCACGGCCATCCTGCTGGGTCTCGTTGT
CCTCTACCATGCCCGGGAGATCCAGCTGTTCATGGTGGACAACGGGGCTGATGACTGGCGCATCG
CCATGACCTGCGAGCGCGTGTTCCTCATCTCGCTAGAGCTGGCAGTGTGCGCCATTCACCCGGTG
CCCGGCCACTACCGCTTCACGTGGACGGCGCGGCTGGCCTTCACGTACGCGCCCTCGGTGGCCGA
GGCCGACGTGGACGTGCTGCTGTCCATCCCCATGTTCCTGCGCCTCTACCTGCTGGGCCGGGTGA
TGCTACTGCACAGCAAAATCTTCACGGACGCCTCGAGCCGCAGCATCGGGGCCCTCAACAAGAT
CACCTTCAACACGCGCTTCGTCATGAAGACACTCATGACCATCTGCCCCGGCACCGTGCTGCTGG
TCTTCAGCATCTCCTCCTGGATCATCGCAGCCTGGACCGTGCGCGTCTGCGAGAGGTACCACGAC
AAGCAGGAAGTGACCAGCAACTTCCTGGGGGCCATGTGGCTGATTTCCATCACCTTCCTCTCCAT

TGGCTACGGCGACATGGTGCCCCACACCTACTGCGGGAAGGGTGTGTGCCTGCTCACTGGCATCA
TGGGAGCTGGCTGTACCGCGCTCGTGGTGGCTGTGGTGGCTCGGAAGCTGGAGCTCACCAAGGCT
GAGAAGCACGTGCACAACTTCATGATGGACACTCAGCTCACCAAGCGGGTAAAAAACGCCGCT
GCTAACGTTCTCAGGGAGACGTGGCTCATCTACAAACATACCAGGCTGGTGAAGAAGCCAGACC
AAGCCCGGGTTCGGAAACACCAGCGTAAGTTCCTCCAAGCCATCCATCAGGCTCAGAAGCTCCG
GAGTGTGAAGATCGAGCAAGGGAAGCTGAACGACCAGGCTAACACGCTTACCGACCTAGCCAA
GACCCAGACCGTCATGTACGACCTTGTATCGGAGCTGCACGCTCAGCACGAGGAGCTGGAGGCC
CGCCTGGCCACCCTGGAAAGCCGCTTGGATGCGCTGGGTGCCTCTCTACAGGCCCTGCCTGGCCT
CATCGCCCAAGCCATACGCCCACCCCCGCCTCCCCTGCCTCCCAGGCCCGGCCCCGGCCCCCAA
GACCAGGCAGCCCGGAGCTCCCCCTGCCGGTGGACGCCCGTGGCCCCCTCGGACTGCGGGTGAC
GGCCCTGCCCGCCACCAGACCCCTAAATCTTGGCCATCGTGTGGCCGCCACCTCCGGGAAGCCTT
GTACAGT

II. **NM_001386974.1** Homo sapiens potassium calcium-activated channel subfamily N member 1 (KCNN1), transcript variant 2, mRNA- **1833 bp**

CCCTCTCCCCTCCAGCCTTGTCCGCCCGCTCGGGCCGAGCCCCGCAGGTCAGTGCAGGAGCCCAG
CCGCTGAGCCATGCCGGGCCCCGGGCGGCCTGCAGCGAGCCCAACCCCTGCACCCAGGTAGTCA
TGAACAGCCACAGCTACAATGGCAGCGTGGGGCGGCCGCTGGGCAGCGGGCCGGGCGCCCTGG
GACGAGACCCTCCGGACCCTGAGGCCGGCCACCCCCCACAACCCCCGCACAGCCCGGGCCTCCA
GGTGGTAGTGGCCAAGAGTGAGCCAGCCCGGCCCTCACCCGGCAGCCCCCGGGGGCAGCCCCA
GGACCAGGACGATGACGAGGATGATGAGGAAGATGAGGCCGGCAGGCAGAGAGCCTCGGGGA
AACCCTCAAATGTGGGCCACCGCCTGGGCCACCGGCGGGCGCTCTTCGAGAAGCGGAAGCGCCT
CAGCGACTATGCCCTCATTTTCGGCATGTTTGGCATCGTCGTCATGGTGACGGAGACCGAGCTGT
CCTGGGGGGTGTACACCAAGGAGTCTCTGTACTCATTCGCACTCAAATGCCTCATCAGCCTCTCC
ACGGCCATCCTGCTGGGTCTCGTTGTCCTCTACCATGCCCGGGAGATCCAGCTGTTCATGGTGGA
CAACGGGGCTGATGACTGGCGCATCGCCATGACCTGCGAGCGCGTGTTCCTCATCTCGCTAGAGC
TGGCAGTGTGCGCCATTCACCCGGTGCCCGGCCACTACCGCTTCACGTGGACGGCGCGGCTGGCC
TTCACGTACGCGCCCTCGGTGGCCGAGGCCGACGTGGACGTGCTGCTGTCCATCCCCATGTTCCT
GCGCCTCTACCTGCTGGGCCGGGTGATGCTACTGCACAGCAAAATCTTCACGGACGCCTCGAGCC
GCAGCATCGGGGCCCTCAACAAGATCACCTTCAACACGCGCTTCGTCATGAAGACACTCATGAC
CATCTGCCCCGGCACCGTGCTGCTGGTCTTCAGCATCTCCTCCTGGATCATCGCAGCCTGGACCGT
GCGCGTCTGCGAGAGGTACCACGACAAGCAGGAAGTGACCAGCAACTTCCTGGGGGCCATGTGG
CTGATTTCCATCACCTTCCTCTCCATTGGCTACGGCGACATGGTGCCCCACACCTACTGCGGGAA
GGGTGTGTGCCTGCTCACTGGCATCATGGGAGCTGGCTGTACCGCGCTCGTGGTGGCTGTGGTGG
CTCGGAAGCTGGAGCTCACCAAGGCTGAGAAGCACGTGCACAACTTCATGATGGACACTCAGCT
CACCAAGCGGGTAAAAAACGCCGCTGCTAACGTTCTCAGGGAGACGTGGCTCATCTACAAACAT
ACCAGGCTGGTGAAGAAGCCAGACCAAGCCCGGGTTCGGAAACACCAGCGTAAGTTCCTCCAA
GCCATCCATCAGGCTCAGAAGCTCCGGAGTGTGAAGATCGAGCAAGGGAAGCTGAACGACCAG
GCTAACACGCTTACCGACCTAGCCAAGACCCAGACCGTCATGTACGACCTTGTATCGGAGCTGC
ACGCTCAGCACGAGGAGCTGGAGGCCCGCCTGGCCACCCTGGAAAGCCGCTTGGATGCGCTGGG
TGCCTCTCTACAGGCCCTGCCTGGCCTCATCGCCCAAGCCATACGCCCACCCCCGCCTCCCCTGC
CTCCCAGGCCCGGCCCCGGCCCCCAAGACCAGGCAGCCCGGAGCTCCCCCTGCCGGTGGACGCC
CGTGGCCCCCTCGGACTGCGGGTGACGGCCCTGCCCGCCACCAGACCCCTAAATCTTGGCCATCG
TGTGGCCGCCACCTCCGGGAAGCCTTGTACAGT

I. **NM_002248.5** ΔEx3-4– **1230 bp**

CGAGGAGACCCCTGCACCCTAGGCTGTTCATGGTGGACAACGGGGCTGATGACTGGCGCATCGC
CATGACCTGCGAGCGCGTGTTCCTCATCTCGCTAGAGCTGGCAGTGTGCGCCATTCACCCGGTGC
CCGGCCACTACCGCTTCACGTGGACGGCGCGGCTGGCCTTCACGTACGCGCCCTCGGTGGCCGAG
GCCGACGTGGACGTGCTGCTGTCCATCCCCATGTTCCTGCGCCTCTACCTGCTGGGCCGGGTGAT
GCTACTGCACAGCAAAATCTTCACGGACGCCTCGAGCCGCAGCATCGGGGCCCTCAACAAGATC
ACCTTCAACACGCGCTTCGTCATGAAGACACTCATGACCATCTGCCCCGGCACCGTGCTGCTGGT
CTTCAGCATCTCCTCCTGGATCATCGCAGCCTGGACCGTGCGCGTCTGCGAGAGGTACCACGACA
AGCAGGAAGTGACCAGCAACTTCCTGGGGGCCATGTGGCTGATTTCCATCACCTTCCTCTCCATT
GGCTACGGCGACATGGTGCCCCACACCTACTGCGGGAAGGGTGTGTGCCTGCTCACTGGCATCAT
GGGAGCTGGCTGTACCGCGCTCGTGGTGGCTGTGGTGGCTCGGAAGCTGGAGCTCACCAAGGCT
GAGAAGCACGTGCACAACTTCATGATGGACACTCAGCTCACCAAGCGGGTAAAAAACGCCGCT
GCTAACGTTCTCAGGGAGACGTGGCTCATCTACAAACATACCAGGCTGGTGAAGAAGCCAGACC
AAGCCCGGGTTCGGAAACACCAGCGTAAGTTCCTCCAAGCCATCCATCAGGCTCAGAAGCTCCG
GAGTGTGAAGATCGAGCAAGGGAAGCTGAACGACCAGGCTAACACGCTTACCGACCTAGCCAA
GACCCAGACCGTCATGTACGACCTTGTATCGGAGCTGCACGCTCAGCACGAGGAGCTGGAGGCC
CGCCTGGCCACCCTGGAAAGCCGCTTGGATGCGCTGGGTGCCTCTCTACAGGCCCTGCCTGGCCT
CATCGCCCAAGCCATACGCCCACCCCCGCCTCCCCTGCCTCCCAGGCCCGGCCCCGGCCCCCAA
GACCAGGCAGCCCGGAGCTCCCCCTGCCGGTGGACGCCCGTGGCCCCCTCGGACTGCGGGTGAC
GGCCCTGCCCGCCACCAGACCCCTAAATCTTGGCCATCGTGTGGCCGCCACCTCCGGGAAGCCTT
GTACAGT

4. **Supplementary Method:**

Western blot

Protein was extracted from subconfluent HEK293 cells (HEK) and HEK293 cells transfected with pcDNA3-*KCNN1* using radioimmunoprecipitation assay (RIPA) buffer (Thermo Fisher, Waltham, USA) and 1% Complete Mini protease inhibitor (Roche, Mannheim, Germany). Total protein concentration was measured using Pierce™ BCA Protein Assay Kit (Thermo Fisher Scientific, Inc., Waltham, MA, USA). Western blots of K_{Ca}2.1 were performed as described previously (Bulk, E. et al., Oncotarget 8, 112268–112282 (2017)). 15 µg of denatured total cellular protein were applied to each lane of protein gels for electrophoresis at 80 mV. After overnight transfer to PVDF membranes at 4 ºC, we detected total amount of protein on the membrane following manufacturer's instructions. Next, we blocked the PVDF membrane with PBS containing 5% skim milk for 1 h, then incubated the blots overnight at 4ºC with primary antibodies against either the N- (HPA065938, 1:200, Atlas Antibodies, Stockholm, Sweden) or the C-terminus (abx029367, 1:200, Abbexa, Cambridge, UK) of the K_{Ca}2.1 protein, recognizing the immunogenic peptide sequences EDDEEDEAGRQRASGKPSNVGHR or KIEQGKLNDQANTLTDLAKTQTVMYDLVSELHAQHEELEARLATLESRLD, respectively. After washing three times with PBS, we applied HRP-conjugated donkey anti-rabbit secondary antibody (1:10000, Donkey Anti-rabbit IgG H&L, #ab6701, Abcam, Cambridge, UK). Chemiluminescence was detected using a Chemidoc MP detection system (Bio-Rad, Hercules, USA), and band intensities were evaluated with the ImageLab software (Bio-Rad, Hercules, USA).

5. **References:**

59. Zhou, Y.; Mokhtari, R.B.; Pan, J.; Cutz, E.; Yeger, H. Carbonic Anhydrase II Mediates Malignant Behavior of Pulmonary Neuroendocrine Tumors. *Am J Respir Cell Mol Biol* **2015**, *52*, doi:10.1165/rcmb.2014-0054OC.

60. Weber, R.; Bertoni, A.P.S.; Bessestil, L.W.; Brasil, B.M.D.A.A.; Brum, L.S.; Furlanetto, T.W. Validation of Reference Genes for Normalization Gene Expression in Reverse Transcription Quantitative PCR in Human Normal Thyroid and Goiter Tissue. *Biomed Res Int* **2014**, *2014*, doi:10.1155/2014/198582.

61. Pigozzi, D.; Ducret, T.; Tajeddine, N.; Gala, J.L.; Tombal, B.; Gailly, P. Calcium Store Contents Control the Expression of TRPC1, TRPC3 and TRPV6 Proteins in LNCaP Prostate Cancer Cell Line. *Cell Calcium* **2006**, *39*, 401–415, doi:10.1016/J.CECA.2006.01.003.

62. Zheng, D.H.; Guo, W.; Sun, F.J.; Xu, G.Z.; Zang, Z. Le; Shu, H.F.; Yang, H. Expression of TRPC6 and BDNF in Cortical Lesions From Patients With Focal Cortical Dysplasia. *J Neuropathol Exp Neurol* **2016**, *75*, 718, doi:10.1093/JNEN/NLW044.

63. Chen, S.J.; Hoffman, N.E.; Shanmughapriy, S.; Bao, L.; Keefer, K.; Conrad, K.; Merali, S.; Takahashi, Y.; Abraham, T.; Hirschler-Laszkiewicz, I.; et al. A Splice Variant of the Human Ion Channel TRPM2 Modulates Neuroblastoma Tumor Growth through Hypoxia-Inducible Factor (HIF)-1/2α. *Journal of Biological Chemistry* **2014**, *289*, doi:10.1074/jbc.M114.620922.

64. O'Grady, S.; Morgan, M.P. Deposition of Calcium in an in Vitro Model of Human Breast Tumour Calcification Reveals Functional Role for ALP Activity, Altered Expression of Osteogenic Genes and Dysregulation of the TRPM7 Ion Channel. *Sci Rep* **2019**, *9*, doi:10.1038/S41598-018-36496-9.

65. Sugimoto, A.; Miyazaki, A.; Kawarabayashi, K.; Shono, M.; Akazawa, Y.; Hasegawa, T.; Ueda-Yamaguchi, K.; Kitamura, T.; Yoshizaki, K.; Fukumoto, S.; et al. Piezo Type Mechanosensitive Ion Channel Component 1 Functions as a Regulator of the Cell Fate Determination of Mesenchymal Stem Cells. *Sci Rep* **2017**, *7*, 17696, doi:10.1038/S41598-017-18089-0.

66. Hu, S.; Li, L.; Huang, W.; Liu, J.; Lan, G.; Yu, S.; Peng, L.; Xie, X.; Yang, L.; Nian, Y.; et al. CAV3.1 Knockdown Suppresses Cell Proliferation, Migration and Invasion of Prostate Cancer Cells by Inhibiting AKT. *Cancer Manag Res* **2018**, *10*, 4603, doi:10.2147/CMAR.S172948.

67. Daniil, G.; Fernandes-Rosa, F.L.; Chemin, J.; Blesneac, I.; Beltrand, J.; Polak, M.; Jeunemaitre, X.; Boulkroun, S.; Amar, L.; Strom, T.M.; et al. CACNA1H Mutations Are Associated With Different Forms of Primary Aldosteronism. *EBioMedicine* **2016**, *13*, 225, doi:10.1016/J.EBIOM.2016.10.002.

68. Chin-Smith, E.C.; Slater, D.M.; Johnson, M.R.; Tribe, R.M. STIM and Orai Isoform Expression in Pregnant Human Myometrium: A Potential Role in Calcium Signaling during Pregnancy. *Front Physiol* **2014**, *5*, doi:10.3389/FPHYS.2014.00169.

69. Elzi, D.J.; Song, M.; Houghton, P.J.; Chen, Y.; Shiio, Y. The Role of FLI-1-EWS, a Fusion Gene Reciprocal to EWS-FLI-1, in Ewing Sarcoma. *Genes Cancer* **2015**, *6*, doi:10.18632/genesandcancer.86.

6. Uncropped Western Blots (Figure S4) and PCR gels (Figure 3)

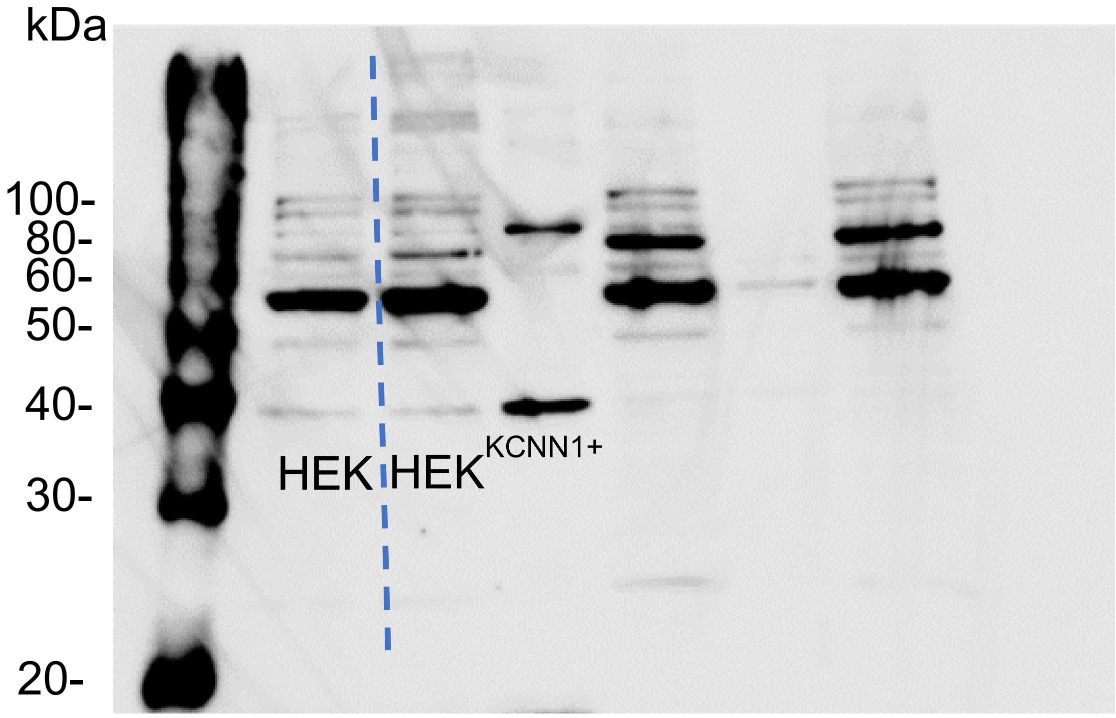

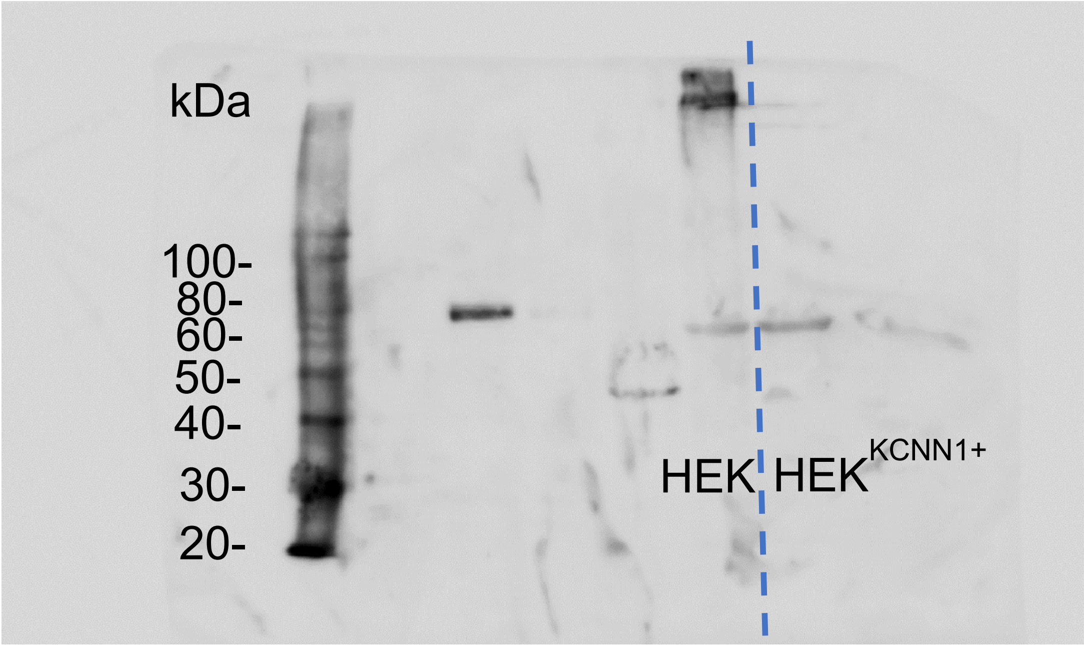

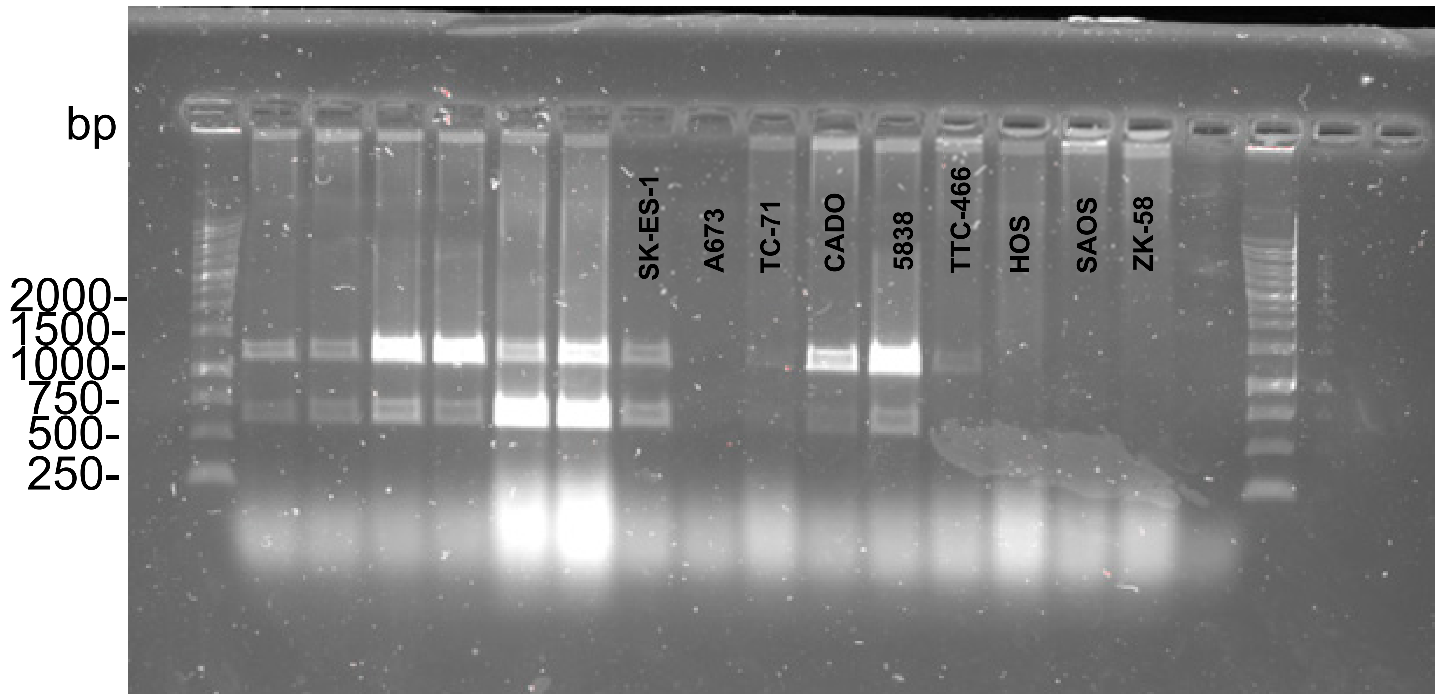
bp
2000-
1500-
1000-
750-
500-
250-
SK-ES-1
A673
TC-71
CADO
5838
TTC-466
HOS
SAOS
ZK-58

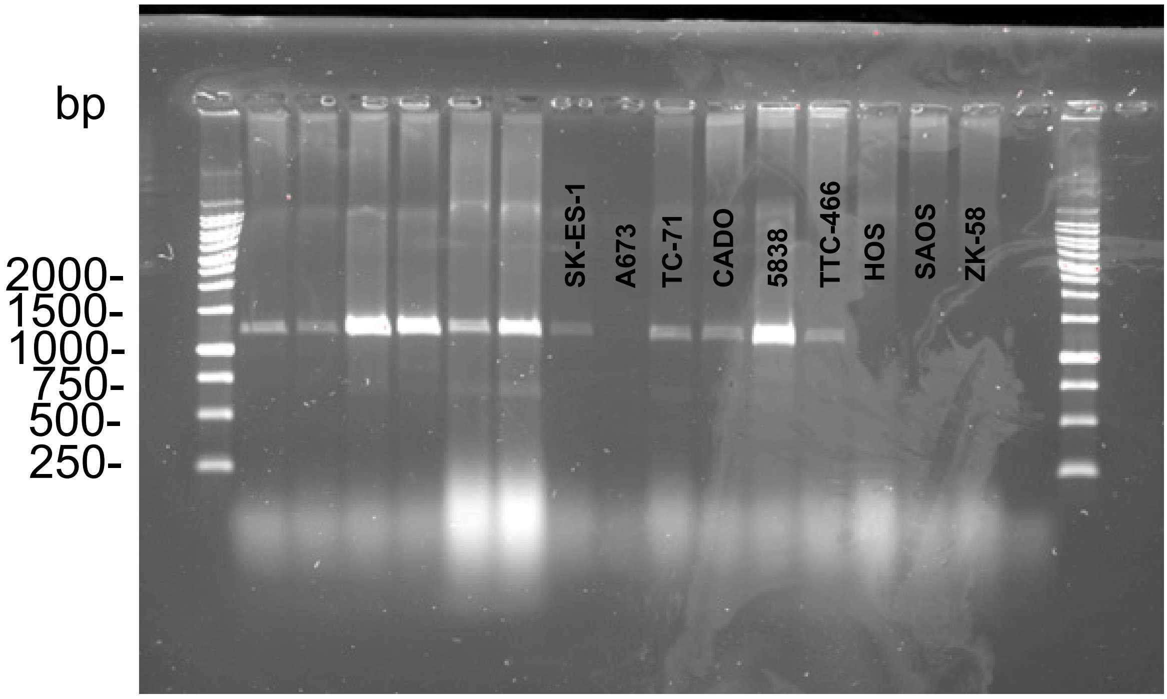
bp
2000-
1500-
1000-
750-
500-
250-
SK-ES-1
A673
TC-71
CADO
5838
TTC-466
HOS
SAOS
ZK-58
